# Macrophage polarization: a key event in the secondary phase of acute spinal cord injury

**DOI:** 10.1111/jcmm.13034

**Published:** 2016-12-13

**Authors:** Xiangyi Kong, Jun Gao

**Affiliations:** ^1^ Department of NeurosurgeryPeking Union Medical College HospitalChinese Academy of Medical SciencesBeijingChina; ^2^ Department of AnesthesiaCritical Care and Pain MedicineMassachusetts General HospitalHarvard Medical SchoolHarvard UniversityBostonMAUSA

**Keywords:** spinal cord injury, inflammation, macrophage, polarization

## Abstract

Acute spinal cord injury (SCI) has become epidemic in modern society. Despite advances made in the understanding of the pathogenesis and improvements in early recognition and treatment, it remains a devastating event, often producing severe and permanent disability. SCI has two phases: acute and secondary. Although the acute phase is marked by severe local and systemic events such as tissue contusion, ischaemia, haemorrhage and vascular damage, the outcome of SCI are mainly influenced by the secondary phase. SCI causes inflammatory responses through the activation of innate immune responses that contribute to secondary injury, in which polarization‐based macrophage activation is a hallmarker. Macrophages accumulated within the epicentre and the haematoma of the injured spinal cord play a significant role in this inflammation. Depending on their phenotype and activation status, macrophages may initiate secondary injury mechanisms and/or promote CNS regeneration and repair. When it comes to therapies for SCI, very few can be performed in the acute phase. However, as macrophage activation and polarization switch are exquisitely sensitive to changes in microenvironment, some trials have been conducted to modulate macrophage polarization towards benefiting the recovery of SCI. Given this, it is important to understand how macrophages and SCI interrelate and interact on a molecular pathophysiological level. This review provides a comprehensive overview of the immuno‐pathophysiological features of acute SCI mainly from the following perspectives: *(i)* the overview of the pathophysiology of acute SCI,* (ii)* the roles of macrophage, especially its polarization switch in acute SCI, and *(iii)* newly developed neuroprotective therapies modulating macrophage polarization in acute SCI.

## Introduction

In recent years, the incidence of spinal cord injury (SCI) increased constantly in population. Despite advances made in the understanding of the pathogenesis and improvements in early recognition and treatment, it remains a devastating event, often producing severe and permanent disability. With a peak incidence in young adults, traumatic SCI remains a costly problem for society; direct medical expenses accrued over the life‐time of one patient range from 500,000 to 2 million US dollars 1.

The mechanisms surrounding injury to the spinal cord itself are often discussed in terms of primary and secondary injury. The primary injury refers to the immediate effect of trauma which includes forces of compression, contusion and shear injury to the spinal cord. A secondary, progressive mechanism of cord injury usually follows, beginning within minutes and evolving over several hours after injury. The processes propagating this phenomenon are complex and incompletely understood. Possible mechanisms include ischaemia, hypoxia, inflammation, oedema, excitotoxicity, disturbances of ion homoeostasis and apoptosis 1. The phenomenon of secondary injury is sometimes clinically manifest by neurologic deterioration over the first 8–12 hrs in patients who initially present with an incomplete cord syndrome.

This article reviews the pathophysiology and several inflammatory cells involved in the SCI. And we focused on the polarization‐based macrophages and newly developed neuroprotective therapies through modulating the switch of polarization.

## The pathophysiology in the secondary phase of SCI

SCI normally takes place in two main stages. The acute stage comes first, in which damage is directly caused by trauma. The sub‐acute stage follows, in which various factors such as excitotoxic damage, haemorrhage, ischaemia and inflammatory changes with subsequent cell necrosis or apoptosis, prolonged Wallerian degeneration and scar formation result in secondary damage to spinal cord tissues. As an innate defensive response, the inflammation response is considered to make a significant contribution to secondary damage after SCI, involving infiltrating macrophages and neutrophils, and central nervous system (CNS)‐resident microglia.

The mechanisms that underlie the onset and progression of secondary injury include breakdown of blood–spinal cord barrier, cellular dysfunction, excitotoxicity, oxidative stress, free radical formation, ischaemia and immune and inflammatory changes 1, 2. These cellular, biochemical and vascular events can be initiated and greatly manipulated by the immune responses to SCI.

A complicated set of interactions and procedures can result in ischaemic conditions. Due to traumatic force, fibrin and platelet thrombi, intravascular coagulation and rupture of post‐capillary venules or sulcal arterioles, venous stasis, distension, proteinaceous fluid leakage and oedema occur. As the pia is relatively firm, the oedema causes increased interstitial pressure, further aggravating the ischaemia. Ischaemia at the primary lesion causes metabolic acidosis owing to the relative anaerobic metabolism, with a resulting decrease in pH, followed by reactive hyperaemia and reperfusion that may promote influx of toxic by‐products including oxygen‐free radicals, resulting in considerable damage to cell structures or oxidative stress 3. Free radicals induce progressive lipid peroxidation in cell membranes, which significantly contributes to neural tissue damage observed in SCI 4. Sources of primordial free radicals in the injured spinal cord include the arachidonic acid cascade, either auto‐oxidation of biogenic amine neurotransmitters, or enzymatic (by‐product of monoamine oxidase‐B activity). Infiltrating macrophages and neutrophils as well as activated microglia offer extra sources of free radicals. Increased free radicals have been linked to a number of aspects of the secondary pathophysiology of acute SCI: increase intracellular Ca^2+^ overload due to decreased activity of Ca^2+^‐ATPase; disruption of mitochondrial dysfunction associated with over‐generation of peroxynitrite (PN), 3‐nitrotyrosine (3‐NT) and 4‐hydroxynonenal (4‐HNE); and impairment of microvascular perfusion as a result of the oxidative damage to spinal microvascular endothelium 5.

Among all the mechanisms of secondary damage, inflammation is the most important, and directly or indirectly controls the sequelae after SCI. The inflammation can be divided into a number of stages: immediate neutrophil stimulation and invasion of resident microglia at 0–2 days, recruitment of blood monocytes to the lesion at 3–7 days and resolution of the scar by anti‐inflammatory macrophages and axonal regrowth from day 7 onward 6. Inflammation has both beneficial and tissue‐damaging effects on SCI. It is obvious that destructive processes such as extracellular matrix deterioration and widespread cell damage can be caused by inflammation. In the first week after SCI, these early inflammatory events also generate a hostile microenvironment for the variety of SCI treatments and therefore create obstacles for transplantation‐oriented therapies. During acute and chronic SCI stages, both systematic and local inflammatory reactions lead to the pathogenesis of neurodegenerative events, forming a cavity and glial scar in the spinal parenchyma and resulting in neuron and glia death. Elimination of the pro‐inflammatory environment in the injured spinal cord has become a major therapeutic target to decrease secondary cell death and facilitate neuronal regeneration. Recent research showed that inflammation is also advantageous to functional recovery and neuronal regeneration 7, 8. Mediated by stimulated macrophages/microglia, inflammation in SCI plays a significant role in clearing deteriorating and damaged tissue.

## Responses of macrophages in SCI

**Figure 1:**
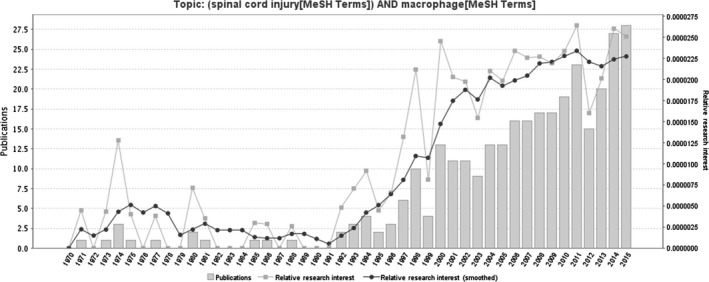
A timeline of the publications related to macrophages and SCI (the date of the end‐point time is 2015).

**Figure 2:**
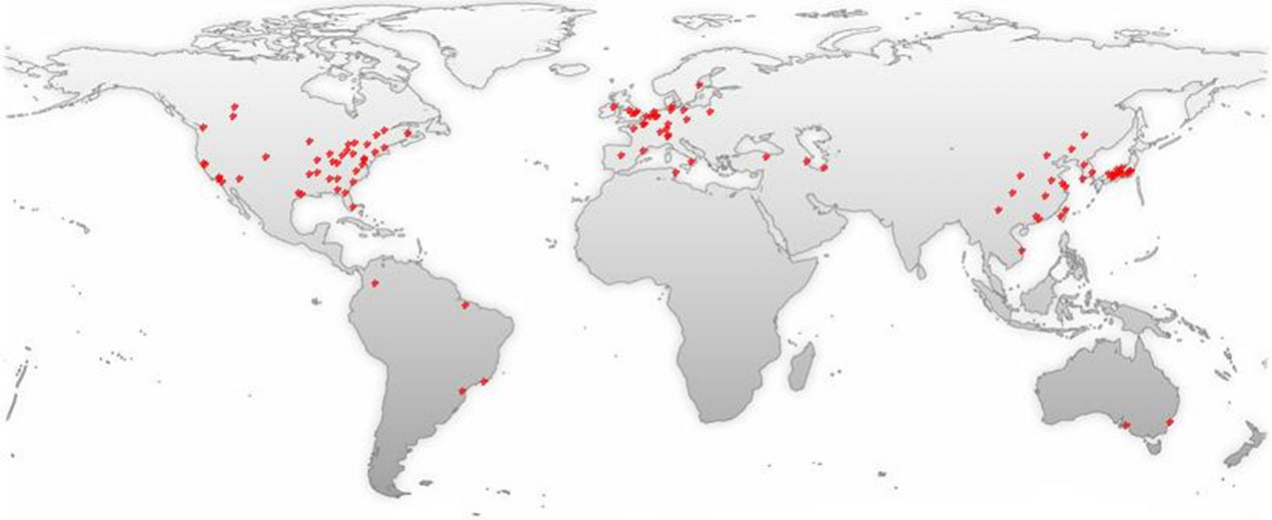
A world map with the global distribution of macrophage‐ and SCI‐related publications based on the analysis of their geolocational data. This figure was generated through GoPubMed (website: http://www.gopubmed.com). GoPubMed is a knowledge‐based search engine for biomedical texts. The technologies used in GoPubMed are generic and can in general be applied to any kind of texts and any kind of knowledge bases. The system was developed at the Technische Universität Dresden by Michael Schroeder and his team at Transinsight. Creation steps for this timeline: import search items to the Search Box at the home page, and then click ‘Statistics’ and download related statistical charts including the timeline and map.

Phagocytes such as macrophages, monocytes, neutrophils and dendritic cells, along with resident microglia, are the major cellular components of the innate non‐specific immune response, which can be immediately stimulated after SCI. After SCI, the collective actions of the non‐specific and adaptive immune system can be recruited and serve various functions which are both neurotoxic and neuroprotective. The number of literatures in relation to macrophage and SCI each year show a general tendency to increase over time. A timeline of the related publications is available as Figure 1. On the basis of a world map with the global distribution of related publications based on the analysis of their geolocational data, the countries that the publications are from are mainly concentrated in Europe, North America and East Asia (Fig. 2).

### Distribution and evolution of macrophages in SCI

**Figure 3:**
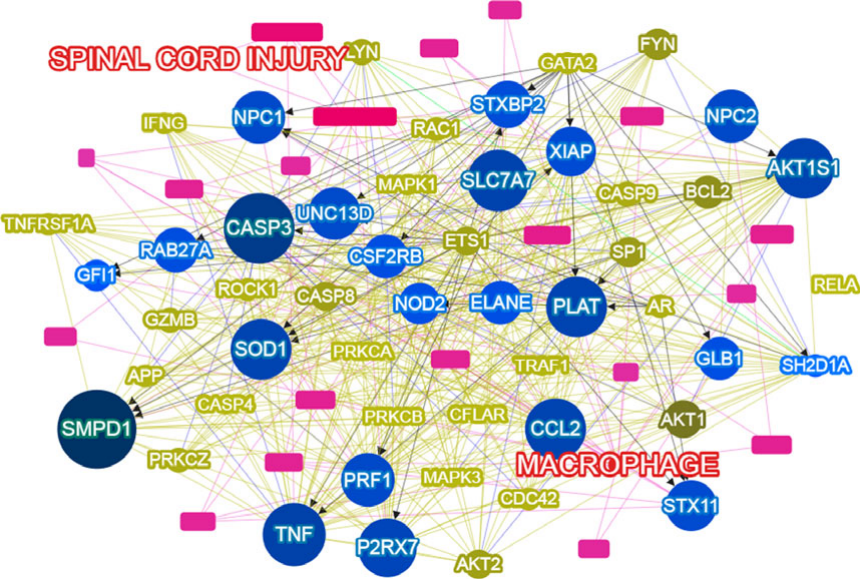
Macrophages interact with lots of phenotype‐based genes and factors during SCI. This figure was generated by virtue of Phenotype‐based Gene Analyzer (Phenolyzer), a tool focusing on discovering genes based on user‐specific disease/phenotype terms. Reference: Yang, Hui, Peter N. Robinson and Kai Wang. Phenolyzer: phenotype‐based prioritization of candidate genes for human diseases. Nature Methods (2015).

Macrophages are heterogeneous and comprised predominantly of two groups: specialized CNS‐resident microglia‐derived macrophages and bone marrow‐derived macrophages. Microglial cells are renewed by local proliferation, arrive in the CNS from yolk sacks in development and are responsible for surveying the CNS parenchyma and aid in synaptic pruning 9, 10. Resident microglia are located in immunizing CNS tissues as mentioned above 9. Macrophages were once believed to play a negative role in the second phase of SCI. Popovich *et al*. 11 showed that reducing the infiltration of macrophages could diminish secondary tissue damage. By making use of the bone marrow chimeras or the expression of Mac‐2 (galactin‐3), infiltrating bone marrow‐derived macrophages can be differentiated from resident microglia‐derived macrophages 12. Shechter *et al*. 13 showed that bone marrow‐derived macrophages are often localized mainly in the margins of the lesion site following SCI, while the resident microglia‐derived macrophages are usually distributed in the lesion core and its margins. After injury, infiltrating bone marrow‐derived macrophages (CX3CR1 low/Mac‐2 high) migrate to the epicentre of injury, while microglia‐derived macrophages (CX3CR1 high/Mac‐2 low) localize to the edges of lesion 14. In other words, the majority of macrophages in the lesion site are bone marrow‐derived macrophages rather than locally activated microglia. These two populations of macrophages with different locations have different functions. Residential microglia‐derived macrophages form a border that seems to seal the lesion and block the spread of damage, whereas bone marrow‐derived macrophages enter the epicentre of injured spinal cord and phagocytize apoptotic and necrotic cells and clear tissue debris such as myelin debris. Using a combination of immunofluorescence, flow cytometry and neuronal tracing techniques, Andrew and Samuel showed that microglia‐derived macrophages contact damaged axons early (24 hrs) after SCI and are the main type of macrophage to contain phagocytic material at day 3 15. Thereafter, infiltrating bone marrow‐derived macrophages become the predominant cells in contact with degenerating axons and contain more phagocytic materials that persist for up to 42 days, which is different from microglia‐derived macrophages 15. Furthermore, after phagocytosis of myelin *in vitro*, bone marrow‐derived macrophages are much more susceptible to apoptotic and necrotic cell death than microglia‐derived macrophages, which has been observed *in vivo* with apoptotic TUNEL‐positive cells of bone marrow‐derived macrophage origin 15. During the pathophysiological procedures of SCI, macrophages with the two separate sources play important roles and interact with lots of phenotype‐based genes and factors such as sphingomyelin phosphodiesterase 1 (SMPD1) and caspase 3 (Fig. 3).

### Subtypes and polarization of macrophages in SCI

In non‐nervous system, studies have shown that activations of different phenotypes of macrophages (macrophage polarization) can lead to cells’ properties of both pro‐inflammation and anti‐inflammation 16. The interferon‐*γ* (IFN‐*γ*) and prototypical T‐helper 1 cytokine (TH1) can activate and induce macrophages to produce cytotoxic mediators (reactive oxygen and nitrogen species) and pro‐inflammatory cytokines (iNOS, IFN‐*γ*, TNF‐*α*, CCL5, IL‐23, IL‐12, IL‐6 and IL‐1*β*), and increase their ability to kill pathogens within cells. By contrast, the IL‐13, IL‐4, TH2, etc. inhibit macrophages from producing pro‐inflammation cytokines 17, 18 and increase their ability to kill extracellular pathogens such as parasite infection 19, 20. Thus, there are two different macrophage phenotypes induced by either TH1 (IFN‐*γ* and TLR signalling) or TH2 (IL‐13 and IL‐4). The former is called M1 or ‘classically activated’ macrophage, while the latter is called M2 or ‘alternatively activated’ macrophage 21, 22, 23, 24. The detailed classification and the characteristics of different subtypes of macrophages are listed in Table 1.

**Table 1:** Characteristics of macrophage subtypes

Classification	M1 (classical)	Ma (alternatively‐activated)			
		M2a	M2b	M2c	M2d
Phenotypes	Classical/Pro‐inflammatory activation	Alternative activation, anti‐inflammatory	Deactivation/wound healing	Repair and remodelling of damaged tissues	Tumour‐associated macrophages (IL‐10^high^ and IL‐12^low^)
Signalling factors	INF‐γ, LPS, TNF‐α, TLR4 signalling	IL‐4 or IL‐13	Immune complexes (binding of FcγR) and IL‐1*β* or LPS	IL‐10, TGF‐*β* or glucocorticoids	IL‐6, LIF and Adenosine
Cytokines	IL‐1, IL‐6, IL‐12, IL‐15, IL‐23 and TNF‐*α*	TGF‐*β*, IL‐10, IL‐1Ra, fibronectin 1, IGF1 and PDGF	IL‐10, TNF‐*α*, IL‐1*β* and IL‐6	Not known	IL‐10, IL‐12, TNF‐*α* and TGF‐*β*
Chemokines	CCL8, CCL 15, CCL 19, CCL20, CXCL9, CXCL 10, CXCL 11 and CXCL 13	CCL13, CCL14, CCL17, CCL18, CCL22, CCL23, CCL24 and CCL26	CCL1, CCL 20, CXCL1, CXCL2 and CXCL3	CCL16, CCL18 and CXCL13	CCL5, CXCL10 and CXCL16
Defining markers	CD16, CD32, CCL2, CD86, MARCO, iNOS	CD206, CD209, argenase‐1, FIZZ1, YM1, IGF‐1, IL1RN	SOCS3, mannose receptor, CD86, TNF‐*α*, CD64	SLAM, Sphk‐1, THBS1, HMOX‐1, CD163	VEGF
Functions in normal healing	Phagocytosis, ability to kill intracellular pathogens by iron restriction, acidification of phagosome and release of ROI and NO	Immunity against parasites, growth stimulation, tissue repair, collagen formation and recruitment of Th2 cells, basophils and eosinophils	Pro‐ and anti‐inflammatory function, B cell class switch and antibody production, recruitment of regulatory T cells (T Reg cells). Phenotype can be induced by clearance of apoptotic neutrophils	Debris scavenging, pro‐healing function and iron sequestration	–
Additional functions in SCI	Causes axon dieback	Remyelination, axon regeneration/reduces dieback	Axon regeneration/reduces dieback	Remyelination	–

In the first days after SCI, levels of some pro‐inflammation cytokines such as IL‐6, IL‐1*β*, TNF‐*α* and NOS are all increased mostly by macrophages 25, 26. Kigerl *et al*. 27 described the polarization of macrophages in detail using mice SCI model and demonstrated that during the early stage of SCI, M1s are in the majority. Quantitative real‐time PCR and cDNA microarray analysis indicated that M1 and M2 biomarkers following SCI rapidly increased. The M2 biomarker, arginase 1, was just transient (returned to the baseline only 7 days after SCI) 27. The expression level of another M2 biomarker, CD206, was obviously higher than in normal spinal cord tissues at 14 days after SCI. In addition, one of the characteristic M1 markers, iNOS, was only transiently increased up to 3 days after injury 27. In contrast, the M1 biomarker CD32 was markedly reduced at 28 days after SCI. It seems that the expressions of iNOS and arginase 1 are reciprocally regulated. This also suggests that after SCI, not all M1 and M2 biomarkers change their expressions in a coordinated way, probably because the regulations of macrophage polarization start at different time‐points and are during different post‐SCI stages, or relative factors in the inflammatory microenvironments favour particular biomarkers and not others. Besides phagocytosis at different time, polarization‐based macrophages also interact with many other pathological processes during SCI, including astrocyte activation 28, oligodendrocyte activation 29 and demyelination 29, 30 (Table 2).

**Table 2:** Interrelationships between macrophages and other pathological processes during spinal cord injury

Crosstalk	Interrelationships
Macrophages and astrocyte activation	The formation of the glial scar by reactive astrocytes is a crucial factor in the potential long‐term recovery of functionality. The infiltration of macrophages to glial scars contributes to axonal diebacks Soluble factors from M1 induce a reactive astrocyte gene expression pattern, while M2 factors inhibit expression of these genes Astrocytes previously stimulated by M2 macrophages are able to decrease macrophage proliferation and activity, and decrease TNF‐*α* production in M1 macrophages, indicating an important role of the astrocyte–macrophage axis in SCI Activation of astrocytes follows and is promoted by the microglial response Inhibition of microglia can reduce damage to oligodendrocytes, inhibit axonal dieback, change the formation of glial scar and improve recovery of locomotive function
Macrophages and oligodendrocyte activation	Oligodendrocytes are injured by macrophages at the lesion epicentre after the injury and continue to undergo apoptosis in the spinal parenchyma for many weeks after SCI Loss of oligodendrocytes leads to demyelination of many spared axons and the loss of conduction of action potential by ascending and descending lateral axons Activated and resting macrophages and microglia secrete molecules such as IL‐1*β*, glutamate, NOS and TNF‐*α* which all contribute to secondary death of oligodendrocyte cells
Macrophages and demyelination	Immunological demyelination is accompanied by a robust activation of macrophage cells without an astrogliosis response Activated macrophages and microglia were reported to exclusively locate to regions of immunological demyelination, with only a few of them outside of the region In spinal lesions during secondary injury after SCI, the activities of microglia and macrophages were significantly higher within regions of immunological demyelination Immunological demyelination creates a unique environment in which astrocytes do not form a glial scar and provides a unique model to understand the putative interaction between astrocytes and activated macrophage/microglial cells M2 macrophages are required for initiation of remyelination

### Classically activated microglia/macrophages (M1) in SCI

It has been proved that many pathophysiological processes during the second phase of SCI, including demyelination and neuronal cell deaths, are strongly linked to macrophages 31, 32, 33, 34, 35 (Table 2). Neuronal loss can be directly mediated by M1 *in vitro* researches 27, 36. Increased amount of M1s expressing iNOS contribute greatly to the tissue damages after SCI, especially during the first 2 weeks 37, 38. Studies showed that chondroitin sulphate proteoglycan, a potential inhibitor of axon growth, is 17‐fold more in M1 than in M2, suggesting that M1 can also suppress the neural regeneration after SCI 39, 40. To some extent, depletion of M1s from the injured spinal cord tissue could preclude the neural retraction and loss induced by repulsive guidance molecule A (RGMA) 41. In 2012, Dalli *et al*. 42 revealed that M1s also express higher levels of prostaglandin and leukotriene B4 (LTB4) than M2s. Leukotrienes, as important bioactive lipids metabolized through 5‐lipoxygenase (5‐LOX) and cyclooxygenase (COX), are able to mediate inflammatory processes in the secondary phase of SCI 43. Inhibiting leukotriene expression by COX/5‐LOX inhibitor licofelone could enhance the anti‐inflammation process within chronic lesions and reduce mechanical hypersensitivities in rats after a few months of SCI 44. In addition, Kigerl *et al*.'s study found that in cell cultures, M2‐conditioned mediums promote long, extensive neurites from dorsal root ganglion neurons, while M1‐conditioned mediums induce short, stunted neurites with multiple branches 27. Hence, M1's detrimental effects in SCI are associated to a variety of targets and factors.

### Alternatively activated macrophages (M2) in SCI

Some researchers believe that M2 can be generated from the phenotypic switch of stimulated microglia/M1 for inflammatory resolution. However, the timing of such generation and their detailed conditions remain unknown. In SCI, M2s, as anti‐inflammatory cells expressing TGF‐*β* and IL‐10, are crucial in reducing pro‐inflammatory milieus induced by CNS glia (resident astrocytes and microglia) and M1s, thus promoting regeneration and neuroprotection of injured spinal cord tissues and promoting the renewal of damaged cells from progenitors. M2s also down‐regulate levels of pro‐inflammatory cytokines and up‐regulate arginase‐1 45. The low numbers of such anti‐inflammatory M2s after SCI prolong the pro‐inflammatory process, thus exerting destructive effects on neural regenerations and neurons’ viabilities 27. *In vitro*, phagocytosis of erythrocytes or myelin by activated macrophages resulted in reduced expressions of pro‐inflammatory cytokines such as IL‐12 and TNF‐*α* 46, 47. So, why macrophages during SCI, which are exposed to large numbers of phagocytosed materials, do not have an anti‐inflammatory phenotype remains unclear. In Kigerl *et al*.'s study, bone marrow‐derived M2s (fluorescence labelled) were transplanted 7 days after SCI into the injured spinal cord; after 3 days, however, transplanted M2s’ percentage decreased by 20–40% 27. But, if M2s were implanted into normal spinal cord tissues, things were different 27. Furthermore, most macrophages in uninjured spinal cord tissues present as M2 phenotypes 48. These findings persuasively indicate the microenvironments of injured spinal cord favours M1 polarization (Table 1). Thus it is interesting to explore whether such microenvironments exist in injured spinal cord or whether injured spinal cord contains particular factors which may preclude M2 polarization.

Three different subsets of M2 macrophages (M2a, M2b and M2c) have been identified 49. They functions differently and are induced in different microenvironments by different polarizing signals (Table 1). M2a and M2c have properties of reparation and anti‐inflammation properties. M2bs have complex abilities to regulate different aspects of inflammatory responses: mostly, they produce large numbers of anti‐inflammatory cytokines and mediate low expression of pro‐inflammatory cytokines; and they can also produce IL‐6, IL‐1*β* and TNF‐*α*. M2cs are able to up‐regulate genes involved in sequestering iron acquired from erythrocyte phagocytosis. In Duluc *et al*.'s study, TAMs represent a novel M2 subset, which was named M2d 50. Heterogeneous M2d populations were found to coexist in the tumour microenvironment (Table 1). MHC‐II^low^ M2d macrophages promote tumour growth, and MHC‐II^high^ M2d macrophages promote tumour inhibition 50, 51.

### Polarization switch of macrophages in SCI

Macrophages have extensive functional plasticity, which allows them to switch from one phenotype to another in the presence of various factors in the inflammatory microenvironment following SCI. M1 macrophages in the injured spinal cord have detrimental effect while M2 macrophages promote a regenerative growth response in adult sensory axons. The predominance of M1 macrophages and lower number of M2 macrophages after SCI may contribute to the secondary damage. However, it remains unclear which factors in the SCI tissue lead to the phenotype switch. Kroner and his colleagues revealed that iron accumulated in macrophages in SCI could increase TNF‐*α* expressions and prevent myelin phagocytosis‐medicated conversion from M1 to M2 52. In Wang *et al*.'s 30 study, myelin debris at the lesion site switches bone marrow‐derived macrophages from M2 towards M1. They primed macrophages to an M2 phenotype with M‐CSF, the cytokine that drives M2 macrophage activation and promotes anti‐inflammatory response and then co‐cultured M2 macrophages with myelin debris to evaluate its effect on macrophage phenotype switch. M2 macrophages expressed high levels of well‐characterized M2 markers including YM1, FIZZ‐1, arginase‐1 and CD206 in the presence of M‐CSF 30. Treatment of M2 macrophages with myelin debris led to a significant decrease in the expression of M2 markers, whereas the level of M1 marker iNOS was markedly increased by myelin debris treatment. In Anhui *et al*.'s study, programmed cell death 1 (PD‐1), a critical immune inhibitory receptor involved in innate and adaptive immune responses, is involved in the modulation of macrophage/microglial polarization 53. In their study, M1‐type macrophages/microglia accumulated in greater numbers in the injured spinal cord of PD‐1 knockout mice. Under polarized stimulation, induced expression of PD‐1 occurred in cultured macrophages and microglia. PD‐1 suppressed M1 polarization by reducing the phosphorylation of signal transducer and activator of transcription 1 (STAT1) and promoted M2 polarization by increasing STAT6 phosphorylation 53. In PD‐1 knockout mice, the M1 response was enhanced *via* the activation of STAT1 and nuclear factor‐kappa B 53. In addition, Lee *et al*. 54 found that endogenous expression of anti‐inflammatory cytokine IL‐4 regulates the extent of acute macrophage activation and the phenotype switch, and confines the ensuing secondary cavity formation after SCI.

### Macrophage activation and SCI healing

As previously described, during SCI, through increased phagocytosis and release of pro‐inflammatory cytokines, M1 macrophages facilitate innate immunity to remove foreign microbes and wound debris from the injury site. M2 macrophages exhibit tissue repair properties, show attenuated production of pro‐inflammatory cytokines and have less reactive oxygen species (ROS) production 55, 56. These mediators allow M2 macrophages to tune inflammatory responses, scavenge debris and promote tissue remodelling and repair. Collectively, this sequential M1–M2 macrophage response results in successful SCI healing. The SCI healing consists of three phases: inflammatory phase, proliferation phase and remodelling phase 57. The features and the roles of macrophages in these three phases are shown in Table 3.

**Table 3:** Features and roles of macrophages in different phases of spinal cord injury healing

Phases	Features and roles of macrophages in different phases of spinal cord injury healing
Inflammatory phase	Triggered by shearing and mechanical damage to cells and spinal cord tissue Disruptions of the blood–spinal cord barrier (BSCB), activation and migration of NG2‐positive, oligodendrocyte precursor cells (OPCs) and swelling of endogenous astrocytes Macrophages during the inflammatory phase of normal wound repair adopt a mixture of M1 and M2a phenotypes M1 macrophages express high levels of TNF‐*α*, IL‐6, IL‐12 and IL‐1*β*; attract neutrophils; boost the inflammatory response to facilitate removal of damaged tissues; and enhance phagocytic abilities that further facilitate debris removal, bacterial removal and sterilization, and elimination of spent neutrophils M2a macrophages initiate the proliferative phase of repair through release of anti‐inflammatory cytokines (IL‐4, CD206 and Fizz‐1), increase cell proliferation and migration *via* release of arginase and Ym1, and promote the beginning of tissue formation through secretion of growth factors
Proliferation phase	Proliferation of glia and neural progenitor cells and induction of scar‐forming astrocytes Macrophages facilitate initial cell proliferation during the later inflammatory phase and maintain proliferation during the proliferative phase then transition cells towards maturation at the end of the proliferative phase to start the remodelling phase Macrophages in the early proliferative phase continue to secrete pro‐inflammatory cytokines but transition towards the release of IL‐10 and some anti‐inflammatory markers Given the mixed pro‐ and anti‐inflammatory cytokines released and the increase in IL‐10 expression, macrophages in the proliferative phase most easily map onto the M2b phenotype During later proliferative stages, the M2b‐mediated IL‐10 release likely stimulates activation of M2c macrophages as evidenced by increased expression of prototypical M2c marker, transforming growth factor‐*β* The key macrophage phenotype regulating the proliferative phase of repair, the M2b macrophage phenotype, is improperly activated after SCI. SCI macrophages do not facilitate proper transitions within the proliferative phase of repair
Remodelling phase	Begins 2–3 weeks after injury and can lasts for months to years Decreased expression of the markers associated with an M2a or M2b phenotype: VEGF, arginase‐1 and IGF‐1 Dominated primarily by M2c macrophages as indicated by high TGF‐*β*, CD163 and CD206 (*i.e*. mannose receptor) expression with concurrent decreases in arginase‐1 M2c macrophages likely play a greater role by inhibiting, rather than promoting, different aspects of the remodelling phase

Proper macrophage‐mediated transitions through the phases of repair are influenced by injury severity, health of the individual, infections and not of least importance, the biochemical milieu and tissue specifics of the injury 57. Changes in the mechanical, cellular and biochemical makeup of the damaged tissue can influence macrophage phenotype and therefore the healing response. Recently, Zhang *et al*. investigated the effect of age on a key indicator of M2 macrophage activation, IL‐10. Following mild–moderate SCI in 4‐ and 14‐month‐old mice, they detected significantly reduced IL‐10 expression with age in the injured spinal cord. Specifically, CD86/IL‐10‐positive M2b macrophages were reduced in 14‐ *versus* 4‐month‐old SCI mice. This age‐dependent shift in macrophage phenotype was associated with impaired SCI healing and functional recovery and enhanced tissue damage in 14‐month‐old SCI mice 58. Their data indicate that age is an important factor that regulates SCI inflammation and recovery even to mild–moderate SCI.

### Macrophage migration inhibitory factor and SCI

Migration inhibitory factor (MIF) is a T cell‐derived, soluble lymphokine. MIF was originally found to inhibit the migration of macrophages and activate them at inflammatory loci 59. Furthermore, MIF functions as a hormone and immune modulator, and as a pro‐inflammatory cytokine, and has been identified in many organs including brain 59. In CNS, MIF is found in astrocytes, ependymal cells and epithelial cells of the choroid plexus. It is elevated acutely in pre‐clinical models of SCI and is neurotoxic to spinal neurons *in vitro* 60. Mice that genetically lacked MIF had enhanced neuronal survival after SCI and more robust functional recovery than wild‐type mice 35. In humans, plasma MIF is elevated in patients with chronic SCI 61. Recently, Bank *et al*. 62 confirmed the hypothesis that MIF is elevated in individuals with acute SCI and correlates with clinical or demographic variables. The main findings of some previous studies 35, 61, 62, 63, 64, 65 about the roles of MIF in SCI are summarized in Table 4.

**Table 4:** Previous studies about roles of macrophage migration inhibitory factor (MIF) in spinal cord injury

Author	Year	Country	Object	Key findings
Fujimoto	1997	Japan	Rats	MIF exists in the rat white matter It is immediately released into the CSF and then re‐synthesized 24 hrs after SCI MIF shows a cell proliferative function on neuronal cells These results suggest that MIF plays an important role for secondary SCI
Koda *et al*.	2004	Japan	Rats	The MIF mRNA was up‐regulated in injured spinal cord, peaking 3 days after SCI MIF was up‐regulated in microglia accumulating in lesion epicentre 3 days after SCI and astrocytes around the cystic cavity 1 week after SCI The MIF may contribute to proliferation of astrocytes around the lesioned site in SCI because of its cell proliferation‐promoting property
Chalimoniuk *et al*.	2006	USA	Mice	Exposure of cultured mouse spinal cord neurons to macrophage migration inhibitory factor markedly increased cellular oxidative stress MIF treatment decreased cell viability, increased cellular lactate dehydrogenase release and induced chromatin condensation and aggregation in cultured spinal cord neurons Exposure to MIF markedly decreased expression and activity of neuronal nitric oxide Macrophage migration inhibitory factor can induce dysfunction of spinal cord neurons, leading to cell death through oxidative stress and intracellular calcium‐dependent pathways
Nishio *et al*.	2008	Japan	Mice	Hind‐limb function recovered significantly in the MIF knockout (KO) mice starting from three weeks after SCI The number of surviving neurons in KO mice was significantly larger than that of WT mice six weeks after injury. The number of NeuN/caspase‐3‐active, double‐positive, apoptotic neurons in the KO mice was significantly smaller than that of the WT mice 24 and 72 hrs after SCI These results suggest that MIF existence hinders neuronal survival after SCI. Suppression of MIF may attenuate detrimental secondary molecular responses of the injured spinal cord
Stein *et al*.	2013	USA	Human	Plasma MIF levels were significantly higher in subjects with SCI than in control subjects Subjects with SCI also exhibited significantly higher plasma levels of monokine induced by interferon‐*γ*/chemokine C‐X‐C motif ligand 9, macrophage colony‐stimulating factor, interleukin‐3 and stem cell growth factor‐*β* (SCGF‐*β*)
Bank *et al*.	2015	USA	Human	MIF was significantly elevated in subjects with acute SCI compared with control subjects after injury Levels of cytokines interleukin‐6, interleukin‐9, interleukin‐16, interleukin‐18, chemokines growth‐related oncogene‐*α*/chemokine (C‐X‐C motif) ligand 1, macrophage inflammatory protein‐1*β*/chemokine (C‐C motif) ligand 4, growth factors hepatocyte growth factor (HGF) and stem cell growth factor‐*β* were also significantly elevated in subjects with acute SCI

## Neuroprotective therapies modulating macrophage polarization in SCI

### Pharmacological techniques for altering SCI macrophage polarization

As mentioned, the ratio of M1:M2 is a significant factor in the repair of SCI. Therefore, increasing the M2 cell population and prolonging the presence of this macrophage subtype in the injured local microenvironment may represent a promising strategy for tissue repair after SCI. Macrophages are plastic and can adopt dynamic phenotypic and functional properties in response to new stimuli 66. Although the pro‐inflammatory SCI environment potentiates M1 phenotype, through pharmacological interventions, it is possible to alter the way macrophages respond to pro‐inflammatory stimuli. To identify non‐invasive, clinically viable, pharmacological techniques for altering SCI macrophage activation, there have been more studies on the effects of certain drugs on SCI, some of which showed strong effects of regulating the polarization and switching the phenotypes of macrophages 66, 67, 68, 69, 70. These pharmacological therapies are shown in Table 5 in detail.

**Table 5:** Some drugs for SCI showing effects of regulating macrophage polarization

Author	Year	Drug	Main findings
Zhang *et al*.	2014	Higenamine	The numbers of CD4 (+) T cells, CD8 (+) T cells, Ly6G (+) neutrophils and CD11b (+) macrophages were all significantly lower in the higenamine‐treated group than that in the control group after SCI Higenamine administration increased the expression of IL‐4 and IL‐10 and promoted M2 macrophage activation Significantly reduced haemoglobin 1 expression was also observed in higenamine‐treated mice with SCI
Li *et al*.	2015	Chemokine Receptor 5 (CCR5) blockade	CCR5 blockade displayed increased myelin sparring and enhanced SC repair process The number of CD4 (+) T cells, CD8 (+) T cells, Ly6G (+) neutrophils and CD11b (+) macrophages were all significantly lower in the anti‐CCR5 group than that in the control group after SCI The IL‐4 and IL‐13 levels in anti‐CCR5 group were markedly higher than that in control group after SCI The anti‐CCR5‐treated group showed increased numbers of arginase‐1‐ or CD206‐expressing macrophages compared with the control IgG group
Zhang *et al*.	2015	Azithromycin	Azithromycin treatment altered macrophage phenotype *in vitro* and lowered the neurotoxic potential of pro‐inflammatory, M1 macrophages
Ji *et al*.	2015	Brain‐derived neurotrophic factor (BDNF)	BDNF could promote the shift of M1 to M2 phenotype and ameliorate the inflammatory microenvironment The roles of BDNF in immunity modulation may enhance neuroprotective effects and partially contribute to the locomotor functional recovery after SCI
Zhang *et al*.	2016	Aldose reductase	Aldose reductase works as a switch which can regulate microglia by polarizing cells to either the M1 or the M2 phenotype under M1 stimulation based on its states of activity

### Hypothermia therapy and hyperbaric oxygen therapy

After SCI, neutrophil influx and morphological indices of microglial activation are reduced by systemic or epidural hypothermia. These changes occur in parallel with improvements in neurological function. In addition, hypothermia may work by reducing cellular metabolic demand, excitotoxicity, vascular permeability, oedema and/or inflammation. A recent Phase I trial showed enhanced conversion rates from American Spinal Injury Association (ASIA) A to B, C or D (~ 43%) without adverse complications in a small cohort of cervical SCI patients who received moderate endovascular cooling 71. Details about the cooling techniques applied to human SCI were published, and a Phase II trial is pending. However, a direct cause–effect relationship has not been confirmed so far.

Hyperbaric oxygen (HBO) therapy is a treatment by which 100% oxygen is administered to a patient at a pressure greater than atmospheric pressure at sea level. HBO therapy promotes healing of damaged tissue, decreases inflammatory response and promotes capillary angiogenesis by increasing tissue oxygen levels by 10–15‐fold. Many studies explored the effects of HBO therapy during SCI and proposed a variety of mechanisms (Table 6), including immunomodulatory effects, downregulating the expression of HMGB1, TLR2, TLR4, NF‐кB, aquaporin 4/9 mRNA, HIF‐1*α*, IL‐6, MMP‐2 and MMP‐9, upregulating VEGF and nitrite levels, affecting the iNOS mRNA–iNOS–NO signalling pathway and suppressing cell apoptosis 72, 73, 74, 75, 76, 77, 78, 79, 80, 81, 82, 83, 84, 85. In Geng *et al*.'s study, HBO therapy was related to considerable increases in the levels of IL‐13 and IL‐4 and decreases in the level of IFN‐*γ* and TNF‐*α*. This was simultaneously related to the levels of M2 macrophages and reduced levels of M1 macrophages 72. The changes were related to functional recovery in the HBO therapy‐transplanted group, related to increased myelin sparing and preserved axons. It can therefore be implied that after SCI HBO therapy changes the inflammation environment by transforming macrophage phenotypes from M1 to M2, this may enhance functional recovery and axonal extension.

**Table 6:** Studies on neuroprotective effects of hyperbaric oxygen therapy on SCI

Author	Year	Subjects	Neuroprotective mechanisms
Lu *et al*.	2012	Rats	HBO intervention can suppress cell apoptosis after SCI
Yang *et al*.	2013	Rats	HBO intervention may reduce the secondary damage of SCI caused by inflammatory responses *via* down‐regulating the expression of HMGB1/NF‐κB and promoting the repair of neurological function
Huang *et al*.	2013	Rats	HBO therapy can promote the neuroprotection following SCI, which may be related to the effect of HBO on the iNOS mRNA–iNOS–NO signalling pathway
Yang *et al*.	2013	Rats	BO reduced SCI‐induced spinal cord oedema, stabilized the blood–spinal cord barrier and promoted recovery of neuronal function by down‐regulating the expression of IL‐6, MMP‐2 and MMP‐9 and up‐regulating the expression of VEGF
Zhou *et al*.	2013	Rats	The elevation and duration of the expression of VEGF and the reduction of expression of HIF‐1*α* by HBO intervention may be inversely related in the repair of damaged spinal cord and neuroprotective effect
Yaman *et al*.	2014	Rats	Increased nitrite levels of SCI rats diminished after HBO treatments compared to the control group
Tan *et al*.	2014	Rats	HBO treatment may mitigate secondary injury to the spinal cord by inhibiting inflammatory responses induced by TLR2/NF‐кB signalling
Long *et al*.	2014	Rats	HBO therapy prevents inflammation apoptosis after SCI, likely through suppression of apoptosis‐associated speck‐like protein and caspase‐3
Wang *et al*.	2014	Rats	HBO therapy reduces apoptosis, down‐regulates aquaporin 4/9 mRNA and protein expression in injured spinal cord tissue, improves the local microenvironment for nerve regeneration, and protects and repairs the spinal cord after injury
Kang *et al*.	2015	Rats	HBO reduced the expressions of HMGB1, TLR4 and NF‐κB and reduced secondary SCI as measured using Basso, Beattie, and Bresnahan scores
Liu *et al*.	2015	Rats	HBO treatment alleviates secondary injury to the spinal cord by inhibiting ER stress‐induced apoptosis, thereby promoting the recovery of neurological function
Hou *et al*.	2015	Rats	HBO therapy played a protective effect on spinal cord injury through reducing apoptosis of neuronal cells and expression of MMP‐9/MMP‐2 gene and protein in rats with SCI
Geng *et al*.	2015	Rats	HBO therapy was associated with significant increases in IL‐4 and IL‐13 levels, and reductions in TNF‐*α* and IFN‐*γ* levels. This was associated simultaneously with the levels of M2 and decreased levels of M1
Sun *et al*.	2016	Rats	HBO treatment enhances autophagy expression in rats after SCI and accelerates cell repair rate

### Transplantation of mesenchymal stem cells

Among the most promising therapeutic approaches for SCI, cell transplantation is a novel one. Adult mesenchymal stem cells derived from the bone marrow (MSC) have been shown to promote anatomical and functional recovery in animal models of SCI by promoting tissue sparing 86, 87, and axonal regeneration 88. The therapeutic benefits of MSC are thought to be primarily related to their secretion of soluble factors and the provision of an extracellular matrix that provides neural protection and support, and secondarily to re‐myelination 89, and neural differentiation 90, 91. MSC transplantation in experimental SCI is reported to enhance tissue preservation after SCI and reduce injury‐induced sensitivity to mechanical stimuli, which is functionally indicative of anti‐inflammatory activity 92. Nakajima *et al*. 93 found that MSC transplantation favoured the development of M2 macrophages, while preventing the development of M1 macrophages, which was accompanied with a decrease in TNF‐*α* and IL‐6, and an increase in IL‐4 and IL‐13. As such, MSC transplantation reduced the size of the SCI site and resulted in less scar tissue formation and increased myelin sparing, correlating with increased axonal growth and improved locomotor function. More specifically, MSC can express or promote secreting a previously unrecognized set of inducers for anti‐inflammatory M2 macrophages: monocyte chemoattractant protein‐1 (MCP‐1) and the secreted ectodomain of sialic acid‐binding Ig‐like lectin‐9 (ED‐Siglec‐9) 94. Depleting MCP‐1 and ED‐Siglec‐9 from the conditioned serum‐free medium from MSC prominently reduced its ability to induce M2 macrophages and to promote functional recovery after SCI, suggesting that it is the MSC‐derived secreted factors that directly convert the pro‐inflammatory conditions prevalent in the damaged spinal cord to tissue‐repairing ones by modulating the phenotype switch of macrophages (M1 to M2) 94.

Limitations with direct MSC transplantation methods preclude effective MSC infusions into post‐SCI treatment protocols. To overcome the limitations, Barminko *et al*. explored the efficacies of alginate micro‐encapsulation in developing implantable vehicles for MSC transplantation. Survivability and secretion functions were kept within the encapsulated MSC population. The MSCs could secret anti‐inflammatory cytokines induced by pro‐inflammatory factors, IFN‐*γ* and TNF‐*α*. Moreover, encapsulated MSC modulated inflammatory macrophage function both *in vivo* and *in vitro*, even without direct MSC–macrophage cells’ contacts, and activated the M2 phenotype.

### M2 macrophage transfer

More directly, in the light of M2 macrophage transfer's application for treating type 1 diabetes, autoimmune encephalomyelitis and chronic inflammatory renal disease in animal models, in a study led by Ma *et al*., 95 M2 macrophage transfer decreased spinal cord lesion volume and resulted in increased myelination of axons and preservation of neurons. This was accompanied by significant locomotor improvement 95. The results indicate that as compared to vehicle treatment or M1 macrophage transfer, M2 adoptive transfer has beneficial effects for the injured spinal cord, in which the increased number of M2 macrophages causes a shift in the immunological response from Th1 to Th2 dominated through the production of anti‐inflammatory cytokines, which in turn induces the polarization of local microglia and/or macrophages to the M2 subtype, and creates a local microenvironment that is conducive to the rescue of residual myelin and neurons and preservation of neuronal function.

## Conclusion

This review has explored the immune pathophysiology of SCI, especially the inflammation dominated by macrophages in the secondary damage. The microenvironment of the injured spinal cord favours M1 polarization with only a transient appearance of M2 macrophages early after SCI. M1 macrophages contribute to the secondary tissue damage and axonal retraction, whereas M2 polarization have protective effects. By deeply understanding how macrophages and their polarization either promote or prevent secondary damage in spinal cord inflammation, some neuroprotective therapies against macrophage polarization in SCI have been developed.

## Funding statement

This study was supported by Peking Union Medical College Youth Research Funds (2016) (Project No. 3332016010; Grant recipient: Xiangyi Kong) and Peking Union Medical College Graduate Student Innovation Fund (2015) (Project No. 2015‐1002‐02‐09; Grant recipient: Xiangyi Kong). The funders had no role in study design, data collection and analysis, decision to publish or preparation of the manuscript.

## Conflict of interest

The authors declare that they have no competing interests.

## Authors' contributions

Xiangyi Kong and Jun Gao both searched the literatures and drafted the article. All authors read and approved the final manuscript.
